# Risks of non-ovarian cancers in women with borderline ovarian tumor: a national cohort study in Sweden

**DOI:** 10.1186/s12885-023-11453-6

**Published:** 2023-10-09

**Authors:** Arturas Dobilas, Filip Jansåker, Xinjun Li, Kristina Sundquist, Christer Borgfeldt

**Affiliations:** 1 Department of Obstetrics and Gynaecology, Skåne University Hospital, Klinikgatan 12, Lund, 221 85 Sweden; 2 Department of Clinical Science Lund, Lund University, Lund, Sweden; 3 Center for Primary Health Care Research, Department of Clinical Sciences Malmö, Lund University, Lund, Sweden; 4 Department of Clinical Microbiology, Rigshospitalet, Copenhagen, Denmark; 5 Center for Community-Based Healthcare Research and Education (CoHRE), Department of Functional Pathology, School of Medicine, Shimane University, Matsue, Japan; 6 Department of Family Medicine and Community Health, Icahn School of Medicine at Mount Sinai, New York, USA; 7 Department of Population Health Science and Policy, Icahn School of Medicine at Mount Sinai, New York, USA

**Keywords:** Borderline ovarian tumors, Cancer risk, Cohort study, Nationwide, Standardized incidence ratios

## Abstract

**Background:**

Associations between different cancer types are known. The affirmation of the risk for non-ovarian cancer after ovarian borderline tumors (BOT) is, however, sparse.

**Aim:**

To analyze the risk of subsequent or simultaneous cancers in women with BOTs compared with the general female Swedish population.

**Methods:**

An open cohort study (1995–2018) was conducted where a diagnosis of BOTs as well as subsequent or simultaneous cancer diagnoses were obtained from the Swedish Cancer Register and matched to the Total Population Register. Each woman with BOT was followed until non-ovarian cancer, death or emigration and could only be included once for the outcome. Standardized incidence ratios (SIRs) with 95% confidence intervals (CIs) for specific non-ovarian cancers were analyzed.

**Results:**

The 4998 women with serous and mucinous BOTs were diagnosed during 1995–2018 with a mean age of 55.7 years (SD 16.0) at diagnosis. Compared with the general female population, women with BOTs had increased risks for non-ovarian cancer in colon (SIR = 2.5; 95% CI 2.0–3.1), rectum (SIR = 1.7; 95% CI 1.1–2.5), small intestine (SIR = 5.0; 95% CI 2.3–9.5), cervix (SIR = 2.5; 95% CI 1.4–4.2), endometrium (SIR = 2.4; 95% CI 1.9–3.1), pancreas (SIR = 2.3; 95% CI 1.4–3.5), upper aerodigestive tract (SIR = 2.2; 95% CI 1.2–3.8), lung (SIR = 1.8; 95% CI 1.4–2.3), kidney (SIR = 2.3; 95% CI 1.4–3.7) and bladder (SIR = 1.8; 95% CI 1.1–2.8). Among women with serous BOTs, the risk of thyroid gland cancer (SIR = 3.1; 95% CI 1.2–6.4) was also increased. Lung and pancreas cancer showed increased risks more than 1 year after a diagnosis of BOT.

**Conclusions:**

This Swedish population-based study demonstrated an increased risk of multiple malignancies including lung and pancreatic cancers beyond the first year of diagnosis in patients with borderline ovarian tumors (BOTs), suggesting a potential shared etiology.

**Supplementary Information:**

The online version contains supplementary material available at 10.1186/s12885-023-11453-6.

## Novelty and impact

The knowledge of non-ovarian cancer after ovarian borderline tumors (BOT) is sparse. This nationwide population-based study found BOTs associations with lung and pancreas cancer more than 1 year after a diagnosis of BOTs. These findings may be used for cancer prevention and surveillance in women diagnosed with BOTs.

## Introduction

Borderline ovarian tumors (BOTs) are a subgroup of the ovarian epithelial tumors, which also are known as atypical proliferating tumors. BOTs have genetically similar changes to their malignant counterparts and are suspected to be precursor lesions to low-grade serous, endometrioid, clear cell, and mucinous ovarian cancer [1]. Most BOTs are serous or mucinous tumors [2] that usually proliferate and show mild–moderate nuclear atypia and mitoses. These changes must be seen in more than 10% of the tumor epithelium and it is acceptable with a stromal micro-invasion of up to 5 mm at the greatest linear measurement in any single focus for a BOT diagnosis [3]. They may spread implants to the peritoneal surfaces and lymph nodes but, in contrast to invasive cancer, do not grow invasively [4, 5]. BOTs are generally diagnosed in an early stage (stage I), confined to one ovary, and have a favorable prognosis [6].

Earlier studies have disclosed higher risks of second primary cancers after ovarian cancer, such as breast cancer, colorectal cancer, bladder cancer, and leukemia [7–9]. Several cancers share common etiology, hereditary factors, and some may be due to chemotherapy-induced secondary malignancies. Some studies have examined risks of non-ovarian cancer following BOTs and found increased risk of colorectal cancer, but decreased the risk for breast cancer compared to patients with a history of ovarian cancer [10–13]. Recently, a large Danish population-based study discovered that serous BOTs were linked with malignant melanoma, thyroid gland cancer, and myeloid leukemia. In contrast, mucinous BOTs were associated with lung cancer, pancreatic cancer, and myeloid leukemia and the results did not differ with the duration of the follow-up period [14]. Women with BOT also seem to have long-term histo-pathological specific increased risks of epithelial ovarian cancer [15].

The main aim of the study was to compare women in Sweden with serous or mucinous BOTs with the general female Swedish population regarding the risk of non-ovarian cancers using the calculated Standardized Incidence Ratio (SIR). We also aimed to examine the risk patterns in relation to age at diagnosis and length of follow-up, adjusted for socioeconomic status and geographical region of residence [16, 17].

## Materials and methods

### Data sources

The Swedish Cancer Register (managed by the National Board of Health and Welfare, in Swedish: *Socialstyrelsen*) was used to collect data on BOTs and outcomes [18]. The Total Population Register (managed by the Swedish governmental agency, *Statistics Sweden*) was used to collect the population of women residing in Sweden during the time period, with data on some of the covariates as well as emigration and death. The register is nearly 100% complete for the entire national population [19]. All linkages between the registry data were performed using the unique 10-digit personal identification number [20] assigned to each person for their lifetime upon birth or immigration but our group had access only to a pseudonymized version of this number to ensure the integrity of all individuals.

### Study design, population, and setting

This was a Swedish nationwide open cohort study conducted between 1995–2018. The total population consisted of 6 838 524 women residing in Sweden with a total follow-up of 116 406 014 person-years (calculated for the total population). Baseline was defined at the first diagnosis of BOTs from 1995 and onwards and the total follow-up time started at baseline and ran until a diagnosis of cancer, death, emigration or end of the follow-up in 2018.

### Ascertainment of the main predictor variable

Serous or mucinous ovarian borderline tumors (BOTs) were obtained from the Swedish Cancer Register [21] starting in 1995 by which time the register had reached full national coverage. Women with a diagnosis of BOT were identified and included during the period 1995–2018 using ICD-7 (topography code 175 with a benign indicator) codes and SNOMED10 (morphology) codes: serous BOTs (84423 and 84513), and mucinous BOTs (84723) [22]. Excluded codes were code 84623 (endometrial and clear cell borderline tumors, *n* = 384) and codes 83801, 83802, and 83811 (*n* = 35) since these tumors were so few for statistical evaluation. Two cases with missing information were excluded.

### Ascertainment of the outcome variables

The outcomes were non-ovarian cancers and were identified in the highly complete nationwide Swedish Cancer Register [18, 21]. This register used the 7^th^ revision of the International Classification of Diseases (ICD-7) (Table S1). Each woman could only be included once for each outcome during the study period.

### Covariates

Age, period (year since BOT diagnosis), highest educational level (as a proxy for socioeconomic status) and geographical region. The rationale for including the covariates were to evaluate if there is any certain time-period (< 1 year, 1–9 years and ≥ 10 years), age, socioeconomical or regional differences among women who later develop non-ovarian cancers. Most cancer occur in older age why the age groups were chosen as < 60, 60–69, and ≥ 70 years of age.

### Statistics

Person-year calculation was started from the first diagnosis of BOTs from 1995 onwards until a diagnosis of non-ovary cancer, death, emigration, or the end of the follow-up in 2018. Standardized incidence ratios were calculated to compare the relative risk of non-ovary cancers in BOTs and in individuals who had never been registered for BOTs. By comparing the observed number of subsequent cancers to the expected number based on population rates, the SIR provides a way to determine whether there is an increased or decreased risk of developing a subsequent cancer compared to the general population. This helps in identifying potential factors that may be associated with the development of subsequent cancers. SIR calculations are recommended and commonly used when the cancers observed are rare diseases and the expected number of cases is small. The SIR was calculated as the ratio of observed (O) to expected (E) number of non-ovary cancer by indirect standardization methods using the following formular:

$$SIR= \frac{{\sum }_{j=1}^{J}{O}_{j}}{\sum_{i=1}^{J}{n}_{j}{\lambda }_{j}^{*}}=\frac{O}{{E}^{*}}$$

where: *O* = ∑*O*_*j*_ denotes the total observed number of non-ovary cancer cases in the study group (registered for BOTs); E* is calculated by applying stratum-specific standard incidence rates (${\lambda }_{j}^{*}$) obtained from the reference group (no registration for BOTs) to the stratum-specific person-year (*n*_*j*_) experience of the study group; *o*_*j*_ represents the observed number of cases that the cohort subjects contribute to the *j*th stratum; and *J* represents the strata defined by the cross-classification of various adjustment variables, including age, educational level, and region [23, 24]. All calculations were standardized by age (5-year-age-groups), period (5-year-period-groups), highest educational level (as a proxy for socioeconomic status) and geographical region. The 95% confidence intervals (CIs) of the SIRs were calculated assuming a Poisson distribution. Concerning multiple comparisons, 99% CIs are shown in footnotes. All of the analyses were performed using SAS software v. 9.4 (SAS Institute Inc., Cary, NC, USA).

## Results

The number of women with BOTs included in the analyses were 2971 women (59%) with serous and 2027 women (41%) with mucinous BOTs, i.e., in total 4998 women diagnosed during 1995–2018 with a mean age of 55.7 years (SD 16.0) at diagnosis. Non-ovarian cancers were found in 526 524 women in the entire female population until 2018 (Supplementary Table S1).

### Subsequent risk of non-ovarian cancer in women with BOTs

Table 1 shows SIRs of non-ovarian cancer sites in all women with serous or mucinous BOTs. The stricter 99% confidence interval, shown in the footnotes, had minor effects on the results. Compared with rates in the general female population, women with BOTs had increased rates of small intestine, colon, endometrial, pancreatic, cervical, lung, kidney, upper aerodigestive tract, bladder, and rectal cancer as well as primary unknown cancer. Women with serous BOTs also had increased rates of both vulvovaginal and thyroid gland cancer. Table S3 shows the risk of cancer after 1 year of follow-up, excluding the first year as a wash-out period. In this analysis women with either histological subgroup (serous and mucinos) BOTs had increased rates of pancreatic, lung and primary unknown cancers.

**Table 1:** Subsequent risks of cancers in women with earlier diagnosis of borderline ovarian tumor (BOT), 1995–2018, sorted by sorted by coding system

Cancer	IDC-7 codes	Total number of events	Serous					Mucinous					All				
			O	E	SIR	95% CI		O	E	SIR	95% CI		O	E	SIR	95% CI	
Upper aerodigestive tract	^a^	7 295	7	3.47	2.02	0.80	4.18	6	2.35	2.55	0.92	5.59	13	5.82	**2.23**	**1.18**	**3.83**
Stomach	151	8 103	6	3.04	1.97	0.71	4.32	2	2.23	0.90	0.08	3.30	8	5.26	1.52	0.65	3.01
Small intestine	152	2 272	5	1.07	**4.67**	**1.47**	**10.99**	**4**	0.74	**5.41**	**1.41**	**13.98**	9	1.81	**4.97**	**2.25**	**9.48**
Colon	153	41 720	52	18.92	**2.75**	**2.05**	**3.61**	29	13.24	**2.19**	**1.47**	**3.15**	81	32.2	**2.52**	**2.00**	**3.13**
Rectum	154	17 450	13	7.71	1.69	0.89	2.89	9	5.42	1.66	0.75	3.17	22	13.1	**1.68**	**1.05**	**2.54**
Liver	155,156	10 183	7	4.41	1.59	0.63	3.29	6	3.13	1.92	0.69	4.20	13	7.54	1.72	0.91	2.96
Pancreas	157	11 593	11	5.67	1.94	0.96	3.48	11	3.9	**2.82**	**1.40**	**5.06**	22	9.58	**2.30**	**1.44**	**3.48**
Lung	162,163	36 128	28	19.44	1.44	0.96	2.08	31	13.18	**2.35**	**1.60**	**3.34**	59	32.6	**1.81**	**1.38**	**2.33**
Breast	170	152 517	75	68.87	1.09	0.86	1.37	59	46.24	1.28	0.97	1.65	134	115	1.16	0.98	1.38
Cervix	171	11 014	8	3.42	**2.34**	**1.00**	**4.63**	7	2.56	**2.73**	**1.08**	**5.67**	15	5.98	**2.51**	**1.40**	**4.15**
Endometrium	172,174	31 922	43	14.76	**2.91**	**2.11**	**3.93**	18	10.21	**1.76**	**1.04**	**2.79**	61	25	**2.44**	**1.87**	**3.14**
Vulvovaginal cancer	176	4 823	6	2.11	**2.84**	**1.02**	**6.23**	1	1.48	0.68	0.00	3.87	7	3.59	1.95	0.77	4.04
Kidney	180	9 538	9	4.32	2.08	0.94	3.97	8	3.01	**2.66**	**1.14**	**5.26**	17	7.33	**2.32**	**1.35**	**3.72**
Bladder	181	13 633	10	6.35	1.57	0.75	2.91	9	4.41	2.04	0.93	3.89	19	10.8	**1.77**	**1.06**	**2.76**
Melanoma	190	27 427	14	12.31	1.14	0.62	1.91	10	8.27	1.21	0.58	2.23	24	20.6	1.17	0.75	1.74
Skin	191	31 693	23	15.27	1.51	0.95	2.26	6	10.38	0.58	0.21	1.27	29	25.7	1.13	0.76	1.62
Nervous system	193	16 798	10	6.54	1.53	0.73	2.82	6	4.46	1.35	0.48	2.95	16	11	1.45	0.83	2.37
Thyroid gland	194	6 534	7	2.27	**3.08**	**1.22**	**6.39**	1	1.57	0.64	0.00	3.65	8	3.84	2.08	0.89	4.13
Endocrine gland	195	13 228	8	5.11	1.57	0.67	3.10	4	3.5	1.14	0.30	2.96	12	8.61	1.39	0.72	2.44
Connective tissue	197	3 013	3	1.1	2.73	0.51	8.07	2	0.79	2.53	0.24	9.31	5	1.89	2.65	0.83	6.22
Primary unknown	199	18 040	16	6.89	**2.32**	**1.32**	**3.78**	13	5.09	**2.55**	**1.35**	**4.38**	29	12	**2.42**	**1.62**	**3.48**
Non-Hodgkins lymphoma	200,202	16 123	8	7.07	1.13	0.48	2.24	8	4.92	1.63	0.69	3.22	16	12	1.33	0.76	2.17
Leukemia	^b^	17 987	11	7.69	1.43	0.71	2.57	5	5.3	0.94	0.30	2.22	16	13	1.23	0.70	2.00
All		526 524	392	235.24	**1.67**	**1.51**	**1.84**	259	161.53	**1.60**	**1.41**	**1.81**	651	397	**1.64**	**1.52**	**1.77**

Bold types: 95% CI does not include 1.00

99% CI for significant SIRs: serous, colon (1.77–4.00); endometrium (1.79–4.39); and all (1.44–1.92); Mucinous, colon (1.19–3.59); lung (1.31–3.80); and all (1.33–1.91); All, small intestine (1.38–11.60); pancreas (1.12–4.03); colon (1.79–3.41); lung (1.20–2.58); cervix (1.01–4.91); endometrium (1.64–3.46); kidney (1.00–4.37); primary unknown (1.31–3.97), and all (1.46–1.83)

*O* Observed, *E* Expected, *SIR* Standardized incidence ratio, *CI* Confidence intervals

^a^140, 141, 143, 144,145,146, 147, 148, 161

^b^204, 205, 207, 208, 209

### Subsequent risks of cancers according to age at diagnosis in women with earlier diagnosis of BOT

Table 2 shows SIRs of non-ovarian cancer sites in all women with serous or mucinous BOTs according to age. Compared with rates in the general female population, women with BOTs who were younger than 60 years of age had increased rates of colon, rectum, lung, endometrium, vulvovaginal and primary unknown cancer. In the age group 60–69 years the risk for upper aerodigestive tract, colon, rectum, pancreas, breast, cervix, endometrium, kidney, melanoma, and primary unknown cancer were increased. In women in the age group 70 years or above the risks were increased for cancer in the upper aerodigestive tract, small intenstine, colon, liver, pancreas, lung, cervix, and kidney. The Supplementary Table S4 shows the SIRs of non-ovarian cancer sites according to age excluding the first year as a wash-out period. The exclusion of the first year after the diagnosis of BOTs showed in women younger than 60 years of age increased risks for lung, vulvovaginal cancer and cancer of primary unknown location. In the age group 60–69 years the risks were increased for colon, lung cancer and melanoma. In the age group 70 years or above the risks for cancer were increased in the small intestine and lung.

**Table 2:** Subsequent risks of cancers according to age at diagnosis in women with earlier diagnosis of borderline ovarian tumor (BOT), 1995–2018, sorted by coding system

Cancer	IDC-7 codes	Total number of events	< 60					60–69					≥ 70				
			O	E	SIR	95% CI		O	E	SIR	95% CI		O	E	SIR	95% CI	
Upper aerodigestive tract	^a^	7 295	0	1.25				6	1.92	**3.13**	**1.12**	**6.85**	7	2.64	**2.65**	**1.05**	**5.49**
Stomach	151	8 103	1	0.76	1.32	0.00	7.54	2	1.33	1.50	0.14	5.53	5	3.18	1.57	0.50	3.70
Small intestine	152	2 272	2	0.32	6.25	0.59	22.99	2	0.55	3.64	0.34	13.37	5	0.94	**5.32**	**1.68**	**12.51**
Colon	153	41 720	19	3.48	**5.46**	**3.28**	**8.54**	25	8.11	**3.08**	**1.99**	**4.56**	37	20.57	**1.80**	**1.27**	**2.48**
Rectum	154	17 450	6	2.06	**2.91**	**1.05**	**6.38**	9	3.97	**2.27**	**1.03**	**4.32**	7	7.11	0.98	0.39	2.04
Liver	155, 156	10 183	3	0.92	3.26	0.61	9.65	2	2.2	0.91	0.09	3.34	8	4.41	1.81	0.77	3.59
Pancreas	157	11 593	2	1.09	1.83	0.17	6.75	9	3.06	**2.94**	**1.33**	**5.61**	11	5.42	**2.03**	**1.01**	**3.64**
Lung	162, 163	36 128	14	4.37	**3.20**	**1.75**	**5.39**	17	11.94	1.42	0.83	2.28	28	16.32	**1.72**	**1.14**	**2.48**
Breast	170	152 517	34	34.46	0.99	0.68	1.38	60	40.45	**1.48**	**1.13**	**1.91**	40	40.19	1.00	0.71	1.36
Cervix	171	11 014	7	3.07	2.28	0.90	4.72	5	1.24	**4.03**	**1.27**	**9.48**	3	1.67	1.80	0.34	5.32
Endometrium	172, 174	31 922	20	4.26	**4.69**	**2.86**	**7.26**	17	8.71	**1.95**	**1.13**	**3.13**	24	12	**2.00**	**1.28**	**2.98**
Vulvovaginal cancer	176	4 823	5	0.55	**9.09**	**2.87**	**21.38**	1	0.87	1.15	0.00	6.59	1	2.16	0.46	0.00	2.65
Kidney	180	9 538	2	1.23	1.63	0.15	5.98	8	2.37	**3.38**	**1.44**	**6.68**	7	3.72	1.88	0.75	3.90
Bladder	181	13 633	4	1.13	3.54	0.92	9.15	2	3	0.67	0.06	2.45	13	6.63	**1.96**	**1.04**	**3.36**
Melanoma	190	27 427	5	7.01	0.71	0.23	1.68	13	5.84	**2.23**	**1.18**	**3.82**	6	7.75	0.77	0.28	1.70
Skin	191	31 693	1	1.72	0.58	0.00	3.33	4	4.54	0.88	0.23	2.28	24	19.38	1.24	0.79	1.85
Nervous system	193	16 798	6	3.93	1.53	0.55	3.35	3	3.73	0.80	0.15	2.38	7	3.34	2.10	0.83	4.34
Thyroid gland	194	6 534	4	1.83	2.19	0.57	5.65	1	0.91	1.10	0.00	6.30	3	1.1	2.73	0.51	8.07
Endocrine gland	195	13 228	5	3.07	1.63	0.51	3.83	5	2.81	1.78	0.56	4.19	2	2.73	0.73	0.07	2.69
Connective tissue	197	3 013	1	0.47	2.13	0.00	12.20	1	0.55	1.82	0.00	10.42	3	0.87	3.45	0.65	10.21
Primary unknown	199	18 040	10	1.55	**6.45**	**3.07**	**11.91**	8	3.07	**2.61**	**1.11**	**5.16**	11	7.36	1.49	0.74	2.68
Non-Hodgkins lymphoma	200, 202	16 123	4	1.97	2.03	0.53	5.25	5	3.47	1.44	0.45	3.39	7	6.55	1.07	0.42	2.21
Leukemia	^b^	17 987	4	2.03	1.97	0.51	5.10	4	3.54	1.13	0.29	2.92	8	7.42	1.08	0.46	2.13
All		526 524	163	84.95	**1.92**	**1.64**	**2.24**	214	121.91	**1.76**	**1.53**	**2.01**	274	189.91	**1.44**	**1.28**	**1.62**

Bold types: 95% CI does not include 1.00

99% CI for significant SIRs: < 60 years, colon (2.48–9.98); endometrium (2.19–8.46); vulvovaginal cancer (2.19–27.22); primary unknown (1.96–14.47); and all (1.52–2.38); 60–69 years, colon (1.58–5.24); and all (1.43–2.12); ≥ 70 years, colon (1.06–2.80); endometrium (1.01–3.43); and all (1.21–1.71)

*O* Observed, *E* Expected, *SIR* Standardized incidence ratio, *CI* Confidence intervals

^a^140, 141, 143, 144,145,146, 147, 148, 161

^b^204, 205, 207, 208, 209

### Subsequent risk of non-ovarian cancer according to length of follow-up

Table 3 shows the risk of the non-ovarian cancer sites according to length of follow-up for women with serous or mucinous BOTs. Within the first year of the diagnosis of BOT increased risks were found for cancer in the upper aerodigestive tract, colon, rectum, small intestine, breast, pancreas, nervous system, bladder, kidney, liver, endometrium, cervix, vulvavagina, and non-Hodgkin lymphoma. The SIRs ≥ 10 years after BOT diagnosis were increased for lung cancer.

**Table 3:** Risks of cancers in women with diagnosis of borderline ovarian tumor (BOT) according to time between BOT diagnosis and non-ovarian cancer diagnosis, 1995–2018, sorted by coding system

Cancer	IDC-7 codes	Total number of events	Follow-up (years)														
			< 1 year					1–9 years					≥ 10 years				
			O	E	SIR	95% CI		O	E	SIR	95% CI		O	E	SIR	95% CI	
Stomach	151	8 103	2	0.27	7.41	0.70	27.24	4	3.36	1.19	0.31	3.08	2	1.63	1.23	0.12	4.51
Small intestine	152	2 272	4	0.09	**44.44**	**11.56**	**114.92**	4	1.1	3.64	0.95	9.40	1	0.62	1.61	0.00	9.25
Colon	153	41 720	39	1.45	**26.90**	**19.12**	**36.80**	25	19.32	1.29	0.84	1.91	17	11.38	1.49	0.87	2.40
Rectum	154	17 450	11	0.63	**17.46**	**8.67**	**31.35**	8	8.12	0.99	0.42	1.95	3	4.38	0.68	0.13	2.03
Liver	155,156	10 183	3	0.37	**8.11**	**1.53**	**24.00**	8	4.6	1.74	0.74	3.44	2	2.57	0.78	0.07	2.86
Pancreas	157	11 593	5	0.44	**11.36**	**3.59**	**26.73**	12	5.69	**2.11**	**1.08**	**3.70**	5	3.45	1.45	0.46	3.41
Lung	162,163	36 128	4	1.46	2.74	0.71	7.08	34	19.34	**1.76**	**1.22**	**2.46**	21	11.82	**1.78**	**1.10**	**2.72**
Breast	170	152 517	19	5.7	**3.33**	**2.00**	**5.22**	76	71.67	1.06	0.84	1.33	39	37.74	1.03	0.73	1.41
Cervix	171	11 014	10	0.34	**29.41**	**14.01**	**54.30**	5	3.93	1.27	0.40	2.99	0	1.71			
Endometrium	172,174	31 922	57	1.22	**46.72**	**35.38**	**60.56**	2	15.45	0.13	0.01	0.48	2	8.29	0.24	0.02	0.89
Vulvovaginal cancer	176	4 823	3	0.16	**18.75**	**3.53**	**55.50**	4	2.16	1.85	0.48	4.79	0	1.27			
Kidney	180	9 538	7	0.36	**19.44**	**7.71**	**40.29**	7	4.48	1.56	0.62	3.24	3	2.49	1.20	0.23	3.57
Bladder	181	13 633	5	0.49	**10.20**	**3.22**	**24.00**	9	6.43	1.40	0.63	2.67	5	3.84	1.30	0.41	3.06
Melanoma	190	27 427	2	0.95	2.11	0.20	7.74	10	12.37	0.81	0.38	1.49	12	7.27	1.65	0.85	2.89
Skin	191	31 693	0	0.95				13	14.36	0.91	0.48	1.55	16	10.34	1.55	0.88	2.52
Nervous system	193	16 798	5	0.58	**8.62**	**2.72**	**20.28**	6	6.94	0.86	0.31	1.89	5	3.49	1.43	0.45	3.37
Thyroid gland	194	6 534	2	0.2	10.00	0.94	36.78	3	2.41	1.24	0.23	3.68	3	1.23	2.44	0.46	7.22
Endocrine gland	195	13 228	2	0.46	4.35	0.41	15.99	6	5.46	1.10	0.40	2.41	4	2.69	1.49	0.39	3.85
Primary unknown	199	18 040	6	0.62	**9.68**	**3.48**	**21.20**	19	7.73	**2.46**	**1.48**	**3.85**	4	3.64	1.10	0.29	2.84
Non-Hodgkins lymphoma	200,202	16 123	5	0.56	**8.93**	**2.82**	**21.00**	8	7.31	1.09	0.47	2.17	3	4.11	0.73	0.14	2.16
Leukemia	^a^	17 987	1	0.6	1.67	0.00	9.55	8	7.84	1.02	0.44	2.02	7	4.55	1.54	0.61	3.19
All		526 524	202	18.84	**10.72**	**9.29**	**12.31**	285	242.42	**1.18**	**1.04**	**1.32**	164	135.51	**1.21**	**1.03**	**1.41**

Bold types: 95% CI does not include 1.00

99% CI for significant SIRs: < 1 year, small intestine (3.74–148.52); pancreas (1.51–34.03); colon (16.06–41.36); rectum (5.71–37.86); breast (1.52–6.09); cervix (8.93–65.99); endometrium (30.80–66.92); kidney (4.17–50.14); bladder (1.36–30.56); nervous system (1.15–25.82); primary unknown (1.71–26.66); non-Hodgkins lymphoma (1.19–26.74); and all (8.69–13.03). 1–9 years, lung (1.01–2.78) and primary unknown (1.12–4.49)

*O* Observed, *E* Expected, *SIR* Standardized incidence ratio, *CI* Confidence intervals

^a^204, 205, 207, 208, 209

## Discussion

In this nationwide study we identified 4998 women with BOTs 1995–2018. These women were found to be at increased risks of lung and pancreas cancer more than 1 year after diagnosis of BOT compared to women in the general population. BOTs were also associated with increased risks of several other cancer types when BOT and the cancers were diagnosed within the same year or later such as colon, rectum, small intestine, cervical, endometrial, kidney, bladder cancer, and cancer of primary unknown origin.

We found a two-fold increase in the risk for lung cancer in women with mucinous BOTs. This was observed in all age groups and in follow up periods between 1–9 years and more than 10 years. This is in line with a similar large Danish population-based register study evaluating the associated cancer risks with earlier BOTs [14]. A smaller study has also reported a slightly increased relative risk of lung cancer in women with earlier BOTs [12]. Several studies have shown an association between smoking and the risk of mucinous ovarian tumors [25, 26]. Additionally, smoking is a risk factor for pancreatic cancer, and we found an increased risk for pancreatic cancer in the group of women older than 60 years [27]. Pancreatic carcinogenesis is influenced by various risk factors, including smoking, obesity, gene mutations. However, without access to detailed data on lifestyle habits, only assumptions about the influence of these risk factors on pancreatic carcinogenesis and BOTs can be made [28]. This study provides additional evidence to substantiate our assertions that pancreatic cancer and BOTs share common risk factors. The risk of pancreatic cancer at the time of BOTs diagnosis and within the initial 1–9 years following BOTs is more than doubled, particularly in cases of mucinous BOTs. The association between mucinous BOTs and lung and pancreatic cancer may be due to similar genetic mutations which has been found for BOTs and ovarian cancer of mucinous histology [29] as well as for lung and pancreatic cancer [26].

We found higher risks of colon and small intestine cancer in women with both serous and mucinous BOTs within the first year of BOT diagnosis. Previous studies have also shown a higher risk of colorectal cancer in women with BOTs [14]. Colorectal cancer has been reported to be more common subsequent to ovarian cancer [9–13]. This has been explained by shared genetics, such as in Lynch syndrome. Lynch syndrome is a condition that increases the risk of many kinds of cancer, affecting one in 300 individuals [30]. Risk might include colon, endometrial and other types of cancer [30, 31]. Our register-based study included a large population, and patients with BOTs were closely monitored over time. The association between mucinous BOTs and pancreatic cancer may partly be explained by the heredity of the Lynch syndrome which is associated with both epithelial ovarian cancer, endometrial, as well as colorectal cancer [32]. Pre-disposition for KRAS and BRAF genetic mutations has been suggested as a possible explanation for the previous findings of increased relative risks of colorectal cancer after BOTs [33]. RAS genes (KRAS, HRAS and NRAS) comprise the most frequently mutated oncogene family in human cancer.

Endometrial and cervical cancer rates were increased within the first year of BOTs diagnosis. Treatment of both endometrial and cervical cancer and/or ovarian cysts with suspicion of BOTs or ovarian malignancy includes hysterectomy and bilateral salpingo-oophorecomy, during which synchronous tumor may be diagnosed incidentally. Moreover, when detailed pathological analysis of the uterus including cervix as well as one or both ovaries has been performed simultaneously existing cancers may be revealed. This may be the case for the increased risk for cervical cancer and borderline ovarian tumors since cervical cancer in more than 99% of cases is due to human papilloma virus (HPV) infections [34]. Endometrial cancer with Lynch syndrome, especially with the MSH2 gene, have shown to have an association with BOT [35].

We found an association between serous BOTs and increased risk for thyroid gland cancer in the total material similarly to the Danish study. Hormonal risk factors and obesity [36], which is a risk factor for both thyroid gland cancer [37] and serous BOTs [38], may contribute to this association. Both serous BOTs [39] and a subset of thyroid gland cancers [40] are associated with BRAF mutations; thus, there is a possibility that women with serous BOTs who subsequently develop thyroid gland cancer may have BRAF mutations.

Our study did not observe increased rates of subsequent breast cancer. This could be explained by the fact that there are other pathways and genetic mutations in breast cancer. This is in contrast to the BRCA gene mutation found in both ovarian and breast cancer [41–44], but there is no found association between BRCA mutations and BOTs. Neither did we find any association between BOTs and myeloid leukemia or malignant melanoma in contrast to our Danish colleagues [14].

### Strengths and limitations

Some of the strengths in this population cohort study is the large number of patients included as well as the complete Swedish Cancer Register [19]. The Danish cohort population study was performed in a similar way showing similar results [14], whereas earlier studies [11, 12] have been much smaller with fewer cases of BOTs. All the major results in this study were based on more than ten observations, the confidence intervals reasonably small and 99% CI were added minimizing the risk of incorrect results of multiple testing and comparisons. Moreover, due to accurate linkage between Swedish registries by use of the unique personal identification number, we had minimal loss of follow-up as well as no recall bias due to the use of clinical diagnoses. Women with borderline ovarian tumors were previously followed-up for several years, which is why there may be a risk for increased numbers of cancers found due to surveillance bias especially during the first years after diagnosis with complimentary diagnostic procedures. Compared with earlier studies we also included confounding factors such as socioeconomic status [16]. However, we cannot fully rule out the risk of residual confounding as certain possible confounders, e.g. obesity or smoking [45–47], were not included. Additionally, the classification of borderline ovarian cancer and tumors has been modified in 2014 [40] and we do not have information about the stage of the BOTs. Therefore, some of the included BOTs with invasive implants in our cohort study may have been classified as low-grade serous carcinoma and this may have affected the results. However, since low-grade ovarian cancer is rare, the probability of this is likely minimal.

## Conclusion

This nationwide population-based study found higher risks of lung and pancreas cancer more than 1 year after diagnosis of BOTs compared to women in the general population. BOTs were also associated with colon, small intestine, cervical, endometrial, and kidney cancer, and cancer of primary unknown origin. These findings could be important for cancer prevention and follow-up in women diagnosed with BOTs. Further studies on the specific causal mechanisms with a specific focus on similar gene mutations behind these associations are needed.

### Supplementary Information

**Additional file 1: Table S1.** Number of cases of cancer (non-ovary cancer) in women in Sweden, 1995-2018. **Table S2.** Study population and Number of cases of cancer (non-ovary cancer) in women in Sweden, 1995-2018. **Table S3.** Subsequent risks of cancers in women with earlier diagnosis of borderline ovarian tumor (BOT), 1995-2018, after 1 year follow-up. **Table S4.** Subsequent risks of cancers by age at diagnosis in women with earlier diagnosis of borderline ovarian tumor (BOT), 1995-2018, after 1 year follow-up.

## Data Availability

This study made use of several national registers and, owing to legal concerns, data cannot be made openly available. Further information regarding the health registries is available from the Swedish National Board of Health and Welfare (https://www.socialstyrelsen.se/en/statistics-and-data/registers/) and Statistics Sweden (https://www.scb.se/en/). The code used in the analysis can be provided upon reasonable request directed to Kristina Sundquist.

## Abbreviations

BOT: Borderline ovarian tumor
SNOMED: Systematized Nomenclature of Medicine
WHO: World Health Organization
ICD: International Classification of Diseases
ICD-7: International Classification of Diseases 7^th^ revision
SIR: Standardized incidence ratio
CI: Confidence interval
HNPCC: Hereditary nonpolyposis colorectal cancer
