# Supplementary material for: Risks of non-ovarian cancers in women with borderline ovarian tumor: a national cohort study in Sweden
Source: BMC Cancer. 2023 Oct 9;23:951. doi: 10.1186/s12885-023-11453-6 (PMC10561436; doi:10.1186/s12885-023-11453-6)
Supplement: Supplementary file 1 — Additional file 1: Table S1. Number of cases of cancer (non-ovary cancer) in women in Sweden, 1995-2018. Table S2. Study population and Number of cases of cancer (non-ovary cancer) in women in Sweden, 1995-2018. Table S3. Subsequent risks of cancers in women with earlier diagnosis of borderline ovarian tumor (BOT), 1995-2018, after 1 year follow-up. Table S4. Subsequent risks of cancers by age at diagnosis in women with earlier diagnosis of borderline ovarian tumor (BOT), 1995-2018, after 1 year follow-up. [file 12885_2023_11453_MOESM1_ESM.docx]

**Supplementary Tables**

**Table S1.** Number of cases of cancer (non-ovary cancer) in women in Sweden, 1995-2018

| **Tumor** | **ICD-7 codes** | **Number of cases** | **%** |
| --- | --- | --- | --- |
| Breast | 170 | 152 517 | 29 |
| Colon | 153 | 41 720 | 7,9 |
| Lung | 162,163 | 36 128 | 6,9 |
| Endometrium | 172, 174 | 31 922 | 6,1 |
| Skin | 191 | 31 693 | 6 |
| Melanoma | 190 | 27 427 | 5,2 |
| Primary unknown | 199 | 18 040 | 3,4 |
| Leukemia | 204, 205, 206, 207, 208, 209 | 17 987 | 3,4 |
| Rectum | 154 | 17 450 | 3,3 |
| Nervous system | 193 | 16 798 | 3,2 |
| Non-Hodgkin’s lymphoma | 200, 202 | 16 123 | 3,1 |
| Bladder | 181 | 13 633 | 2,6 |
| Endocrine gland | 195 | 13 228 | 2,5 |
| Pancreas | 157 | 11 593 | 2,2 |
| Cervix | 171 | 11 014 | 2,1 |
| Liver | 155, 156 | 10 183 | 1,9 |
| Kidney | 180 | 9 538 | 1,8 |
| Stomach | 151 | 8 103 | 1,5 |
| Upper aerodigestive tract | 140, 141, 143, 144, 145, 146, 147, 148, 161 | 7 295 | 1,4 |
| Thyroid gland | 194 | 6 534 | 1,2 |
| Myeloma | 203 | 6 190 | 1,2 |
| Vulvovaginal cancer | 176 | 4 823 | 0,9 |
| Connective tissue | 197 | 3 013 | 0,6 |
| Esophagus | 150 | 2 505 | 0,5 |
| Small intestine | 152 | 2 272 | 0,4 |
| Anus | 1541 | 2 127 | 0,4 |
| Hodgkin’s disease | 201 | 1 961 | 0,4 |
| Others |  | 1 552 | 0,3 |
| Eye | 192 | 1 387 | 0,3 |
| Salivary gland | 142 | 1 033 | 0,2 |
| Bone | 196 | 735 | 0,1 |
| **All** |  | **526 524** | **100** |

| **Table S2.** Study population and Number of cases of cancer (non-ovary cancer) in women in Sweden, 1995-2018 | | | | | |
| --- | --- | --- | --- | --- | --- |
|  | Population | |  | Number of cases | |
|  | No | % |  | No. | % |
| Total | 683 8524 |  |  | 526 524 |  |
| Educational level |  |  |  |  |  |
| <12 years | 4 469 273 | 65.4 |  | 363 043 | 69.0 |
| ≥ 12 years | 2 369 251 | 34.6 |  | 163 481 | 31.0 |
| Region of residence |  |  |  |  |  |
| Large cities | 3 679 139 | 53.8 |  | 265 667 | 50.5 |
| Others | 3 159 385 | 46.2 |  | 260 857 | 49.5 |

**Table S3.** Subsequent risks of cancers in women with earlier diagnosis of borderline ovarian tumor (BOT), 1995-2018, after one year follow-up.

|  |  |  | **Serous** |  |  |  |  |  | **Mucinous** |  |  |  |  |  |  | **All** |  |  |
| --- | --- | --- | --- | --- | --- | --- | --- | --- | --- | --- | --- | --- | --- | --- | --- | --- | --- | --- |
| **Cancer** | **IDC-7 codes** | **O** | **E** | **SIR** | **95% CI** | |  | **O** | **E** | **SIR** | **95% CI** | |  | **O** | **E** | **SIR** | **95% CI** | |
| Upper aerodigestive tract | a) | 6 | 3.31 | 1.81 | 0.65 | 3.97 |  | 4 | 2.25 | 1.78 | 0.46 | 4.6 |  | 10 | 5.56 | 1.8 | 0.86 | 3.32 |
| Stomach | 151 | 4 | 2.88 | 1.39 | 0.36 | 3.59 |  | 2 | 2.11 | 0.95 | 0.09 | 3.49 |  | 6 | 4.99 | 1.2 | 0.43 | 2.63 |
| Small intestine | 152 | 2 | 1.02 | 1.96 | 0.18 | 7.21 |  | 3 | 0.71 | 4.23 | 0.8 | 12.51 |  | 5 | 1.73 | 2.89 | 0.91 | 6.8 |
| Colon | 153 | 26 | 18.05 | 1.44 | 0.94 | 2.11 |  | 16 | 12.66 | 1.26 | 0.72 | 2.06 |  | 42 | 30.71 | 1.37 | 0.99 | 1.85 |
| Rectum | 154 | 4 | 7.33 | 0.55 | 0.14 | 1.41 |  | 7 | 5.17 | 1.35 | 0.54 | 2.81 |  | 11 | 12.5 | 0.88 | 0.44 | 1.58 |
| Liver | 155,156 | 5 | 4.19 | 1.19 | 0.38 | 2.81 |  | 5 | 2.98 | 1.68 | 0.53 | 3.95 |  | 10 | 7.17 | 1.39 | 0.66 | 2.57 |
| Pancreas | 157 | **8** | **5.41** | **1.48** | **0.63** | **2.93** |  | **9** | **3.72** | **2.42** | **1.1** | **4.61** |  | **17** | **9.13** | **1.86** | **1.08** | **2.99** |
| Lung | 162,163 | **26** | **18.54** | **1.4** | **0.92** | **2.06** |  | **29** | **12.62** | **2.3** | **1.54** | **3.3** |  | **55** | **31.16** | **1.77** | **1.33** | **2.3** |
| Breast | 170 | 62 | 65.37 | 0.95 | 0.73 | 1.22 |  | 53 | 44.05 | 1.2 | 0.9 | 1.57 |  | 115 | 109.42 | 1.05 | 0.87 | 1.26 |
| Cervix | 171 | 1 | 3.22 | 0.31 | 0 | 1.78 |  | 4 | 2.42 | 1.65 | 0.43 | 4.27 |  | 5 | 5.64 | 0.89 | 0.28 | 2.09 |
| Endometrium | 172,174 | 3 | 14.02 | 0.21 | 0.04 | 0.63 |  | 1 | 9.73 | 0.1 | 0 | 0.59 |  | 4 | 23.75 | 0.17 | 0.04 | 0.44 |
| Vulvovaginal cancer | 176 | 3 | 2.01 | 1.49 | 0.28 | 4.42 |  | 1 | 1.41 | 0.71 | 0 | 4.07 |  | 4 | 3.42 | 1.17 | 0.3 | 3.02 |
| Kidney | 180 | 5 | 4.11 | 1.22 | 0.38 | 2.86 |  | 5 | 2.87 | 1.74 | 0.55 | 4.1 |  | 10 | 6.98 | 1.43 | 0.68 | 2.64 |
| Bladder | 181 | 8 | 6.06 | 1.32 | 0.56 | 2.61 |  | 6 | 4.22 | 1.42 | 0.51 | 3.12 |  | 14 | 10.28 | 1.36 | 0.74 | 2.29 |
| Melanoma | 190 | 12 | 11.73 | 1.02 | 0.53 | 1.79 |  | 10 | 7.9 | 1.27 | 0.6 | 2.34 |  | 22 | 19.63 | 1.12 | 0.7 | 1.7 |
| Skin | 191 | 23 | 14.69 | 1.57 | 0.99 | 2.35 |  | 6 | 10.01 | 0.6 | 0.22 | 1.31 |  | 29 | 24.7 | 1.17 | 0.79 | 1.69 |
| Nervous system | 193 | 6 | 6.19 | 0.97 | 0.35 | 2.12 |  | 5 | 4.23 | 1.18 | 0.37 | 2.78 |  | 11 | 10.42 | 1.06 | 0.52 | 1.9 |
| Thyroid gland | 194 | 5 | 2.15 | 2.33 | 0.73 | 5.47 |  | 1 | 1.49 | 0.67 | 0 | 3.85 |  | 6 | 3.64 | 1.65 | 0.59 | 3.61 |
| Endocrine gland | 195 | 7 | 4.83 | 1.45 | 0.57 | 3 |  | 3 | 3.32 | 0.9 | 0.17 | 2.67 |  | 10 | 8.15 | 1.23 | 0.58 | 2.27 |
| Connective tissue | 197 | 3 | 1.04 | 2.88 | 0.54 | 8.54 |  | 1 | 0.75 | 1.33 | 0 | 7.64 |  | 4 | 1.79 | 2.23 | 0.58 | 5.78 |
| Primary unknown | 199 | **14** | **6.53** | **2.14** | **1.17** | **3.61** |  | **9** | **4.83** | **1.86** | **0.84** | **3.55** |  | **23** | **11.36** | **2.02** | **1.28** | **3.04** |
| Non-Hodgkins lymphoma | 200,202 | 6 | 6.73 | 0.89 | 0.32 | 1.95 |  | 5 | 4.69 | 1.07 | 0.34 | 2.51 |  | 11 | 11.42 | 0.96 | 0.48 | 1.73 |
| Myeloma | 203 | 3 | 2.68 | 1.12 | 0.21 | 3.31 |  | 1 | 1.89 | 0.53 | 0 | 3.03 |  | 4 | 4.57 | 0.88 | 0.23 | 2.26 |
| Leukemia | b) | 11 | 7.33 | 1.5 | 0.74 | 2.69 |  | 4 | 5.06 | 0.79 | 0.21 | 2.04 |  | 15 | 12.39 | 1.21 | 0.68 | 2 |
| All | | **257** | **223.82** | **1.15** | **1.01** | **1.3** |  | **192** | **154.11** | **1.25** | **1.08** | **1.44** |  | **449** | **377.93** | **1.19** | **1.08** | **1.3** |
| O=Observed; E=Expected; SIR=Standardized incidence ratio; CI=Confidence intervals. Bold types: 95% CI does not include 1.00. Cancer types with less than 4 subsequent cancers are not shown | | | | | | | | | | | | | | | |  |  |  |
| a) = 140, 141, 143, 144,145,146, 147, 148, 161 b) = 204, 205, 207, 208, 209 | | | |  |  |  |  |  |  |  |  |  |  |  |  |  |  |  |
|  |  |  |  |  |  |  |  |  |  |  |  |  |  |  |  |  |  |  |

**Table S4.** Subsequent risks of cancers by age at diagnosis in women with earlier diagnosis of borderline ovarian tumor (BOT), 1995-2018, after one year follow-up.

|  |  |  |  | <60 |  |  |  | |  | | 60-69 | |  |  |  |  |  |  | >70 |  |  |
| --- | --- | --- | --- | --- | --- | --- | --- | --- | --- | --- | --- | --- | --- | --- | --- | --- | --- | --- | --- | --- | --- |
| **Cancer** | **IDC-7 codes** | **O** | **E** | **SIR** | **95% CI** | |  | | **O** | | **E** | | **SIR** | **95% CI** | |  | **O** | **E** | **SIR** | **95% CI** | |
| Upper aerodigestive tract | a) | 0 | 1.18 |  |  |  |  | | 5 | | 1.84 | | 2.72 | 0.86 | 6.39 |  | 5 | 2.53 | 1.98 | 0.62 | 4.65 |
| Stomach | 151 | 1 | 0.71 | 1.41 | 0 | 8.07 |  | | 1 | | 1.26 | | 0.79 | 0 | 4.55 |  | 4 | 3.03 | 1.32 | 0.34 | 3.41 |
| Small intestine | 152 | 1 | 0.3 | 3.33 | 0 | 19.11 |  | | 0 | | 0.52 | |  |  |  |  | **4** | **0.91** | **4.4** | **1.14** | **11.37** |
| Colon | 153 | 6 | 3.26 | 1.84 | 0.66 | 4.03 |  | | **15** | | **7.73** | | **1.94** | **1.08** | **3.21** |  | 21 | 19.7 | 1.07 | 0.66 | 1.63 |
| Rectum | 154 | 2 | 1.93 | 1.04 | 0.1 | 3.81 |  | | 6 | | 3.78 | | 1.59 | 0.57 | 3.48 |  | 3 | 6.79 | 0.44 | 0.08 | 1.31 |
| Liver | 155,156 | 3 | 0.87 | 3.45 | 0.65 | 10.21 |  | | 2 | | 2.1 | | 0.95 | 0.09 | 3.5 |  | 5 | 4.21 | 1.19 | 0.37 | 2.79 |
| Pancreas | 157 | 1 | 1.03 | 0.97 | 0 | 5.57 |  | | 7 | | 2.91 | | 2.41 | 0.95 | 4.98 |  | 9 | 5.2 | 1.73 | 0.78 | 3.3 |
| Lung | 162,163 | **13** | **4.1** | **3.17** | **1.68** | **5.44** |  | | 17 | | 11.4 | | 1.49 | 0.87 | 2.39 |  | **25** | **15.66** | **1.6** | **1.03** | **2.36** |
| Breast | 170 | 25 | 32.31 | 0.77 | 0.5 | 1.14 |  | | **53** | | **38.58** | | **1.37** | **1.03** | **1.8** |  | 37 | 38.52 | 0.96 | 0.68 | 1.33 |
| Cervix | 171 | 4 | 2.86 | 1.4 | 0.36 | 3.62 |  | | 1 | | 1.18 | | 0.85 | 0 | 4.86 |  | 0 | 1.59 |  |  |  |
| Endometrium | 172,174 | 0 | 3.98 |  |  |  |  | | 1 | | 8.28 | | 0.12 | 0 | 0.69 |  | 3 | 11.48 | 0.26 | 0.05 | 0.77 |
| Vulvovaginal cancer | 176 | **3** | **0.51** | **5.88** | **1.11** | **17.41** |  | | 1 | | 0.83 | | 1.2 | 0 | 6.91 |  | 0 | 2.07 |  |  |  |
| Kidney | 180 | 1 | 1.15 | 0.87 | 0 | 4.98 |  | | 5 | | 2.26 | | 2.21 | 0.7 | 5.2 |  | 4 | 3.55 | 1.13 | 0.29 | 2.91 |
| Bladder | 190 | 3 | 1.06 | 2.83 | 0.53 | 8.38 |  | | 2 | | 2.86 | | 0.7 | 0.07 | 2.57 |  | 9 | 6.36 | 1.42 | 0.64 | 2.7 |
| Melanoma | 190 | 4 | 6.59 | 0.61 | 0.16 | 1.57 |  | | **12** | | **5.59** | | **2.15** | **1.1** | **3.76** |  | 6 | 7.45 | 0.81 | 0.29 | 1.76 |
| Skin | 191 | 1 | 1.63 | 0.61 | 0 | 3.52 |  | | 4 | | 4.36 | | 0.92 | 0.24 | 2.37 |  | 24 | 18.72 | 1.28 | 0.82 | 1.91 |
| Nervous system | 193 | 3 | 3.67 | 0.82 | 0.15 | 2.42 |  | | 1 | | 3.55 | | 0.28 | 0 | 1.61 |  | 7 | 3.2 | 2.19 | 0.87 | 4.53 |
| Thyroid gland | 194 | 4 | 1.71 | 2.34 | 0.61 | 6.05 |  | | 1 | | 0.87 | | 1.15 | 0 | 6.59 |  | 1 | 1.05 | 0.95 | 0 | 5.46 |
| Endocrine gland | 195 | 3 | 2.88 | 1.04 | 0.2 | 3.08 |  | | 5 | | 2.69 | | 1.86 | 0.59 | 4.37 |  | 2 | 2.6 | 0.77 | 0.07 | 2.83 |
| Connective tissue | 197 | 1 | 0.44 | 2.27 | 0 | 13.03 |  | | 1 | | 0.52 | | 1.92 | 0 | 11.02 |  | 2 | 0.83 | 2.41 | 0.23 | 8.86 |
| Primary unknown | 199 | **9** | **1.45** | **6.21** | **2.81** | **11.83** |  | | 6 | | 2.9 | | 2.07 | 0.74 | 4.53 |  | 8 | 7.01 | 1.14 | 0.49 | 2.26 |
| Non-Hodgkins lymphoma | 200,202 | 3 | 1.85 | 1.62 | 0.31 | 4.8 |  | | 3 | | 3.31 | | 0.91 | 0.17 | 2.68 |  | 5 | 6.27 | 0.8 | 0.25 | 1.88 |
| Myeloma | 203 | 1 | 0.61 | 1.64 | 0 | 9.4 |  | | 2 | | 1.3 | | 1.54 | 0.15 | 5.66 |  | 1 | 2.68 | 0.37 | 0 | 2.14 |
| Leukemia | b) | 4 | 1.91 | 2.09 | 0.54 | 5.42 |  | | 4 | | 3.38 | | 1.18 | 0.31 | 3.06 |  | 7 | 7.11 | 0.98 | 0.39 | 2.04 |
| All |  | **98** | **79.7** | **1.23** | **1** | **1.5** |  | | **156** | | **116.25** | | **1.34** | **1.14** | **1.57** |  | 195 | 182 | 1.07 | 0.93 | 1.23 |
| O=Observed; E=Expected; SIR=Standardized incidence ratio; CI=Confidence intervals. Bold types: 95% CI does not include 1.00. Cancer types with less than 4 subsequent cancers are not shown | | | | | | | | | | | | | |  |  |  |  |  |  |  |  |
| a) = 140, 141, 143, 144,145,146, 147, 148, 161 b) = 204, 205, 207, 208, 209 | | | | | | | |  | |  | |  | |  |  |  |  |  |  |  |  |
|  |  |  |  |  |  |  |  |  | |  | |  | |  |  |  |  |  |  |  |  |
